# Pathologic Etiologies of Late and Very Late Stent Thrombosis following First-Generation Drug-Eluting Stent Placement

**DOI:** 10.1155/2012/608593

**Published:** 2012-11-21

**Authors:** Fumiyuki Otsuka, Masataka Nakano, Elena Ladich, Frank D. Kolodgie, Renu Virmani

**Affiliations:** Department of Cardiovascular Pathology, CVPath Institute Inc., 19 Firstfield Road, Gaithersburg, MD 20878, USA

## Abstract

Several randomized and observational studies have reported steady increase in cumulative incidence of late and very late ST (LST/VLST) following first-generation drug-eluting stents (DES: sirolimus-(SES) and paclitaxel-(PES)) up to 5 years. Pathologic studies have identified uncovered struts as the primary substrate responsible for LST/VLST following DES, where delayed arterial healing is associated with stent struts penetrating into the necrotic core, long/overlapping stents, and bifurcation stenting especially in flow divider region. Grade V stent fracture also induces LST/VLST and restenosis. Hypersensitivity reaction is exclusive to SES as an etiology of LST/VLST, whereas malapposition secondary to excessive fibrin deposition is associated with PES. Uncovered struts can be identified in SES and PES with duration of implant beyond 12 months, particularly in stents placed for “off-label” indications. Neoatherosclerosis is another important contributing factor for VLST in DES and bare metal stents (BMS); however, DES shows rapid and more frequent development of neoatherosclerosis than BMS. Future pathologic studies should address the long-term safety of newer generation DES including zotarolimus- and everolimus-eluting stents in terms of the improvement in reendothelialization, decreased inflammation and fibrin deposition as well as a lower incidence of stent fracture-related adverse events, and reduced neoatherosclerosis, which likely contribute to the decreased risk of LST/VLST and better patient outcomes.

## 1. Introduction

Percutaneous coronary interventions (PCI) involving stenting are the most widely performed procedures for the treatment of symptomatic coronary disease [1]. Drug-eluting stents (DES) have dramatically reduced restenosis rates and have become the standard of care for the treatment of atherosclerotic coronary artery disease [2–4]. However, concern still exists about the long-term safety of DES technology since several randomized and observational studies have shown a steady increase in cumulative incidence of very late stent thrombosis (ST) associated with first-generation DES (sirolimus-(SES) and paclitaxel-eluting stents (PES)) up to 5 years [5–9], while pathologic studies have suggested delayed re-endothelialization as an important substrate [10, 11]. More recently, the development of atherosclerotic changes within the neointima (neoatherosclerosis) has been identified as another important mechanism of very late ST [12]. DESs have been implanted in millions of patients worldwide; therefore, understanding the histopathologic findings following deployment of such devices in patients is of paramount importance. This paper will focus on the pathologic mechanisms of late and very late ST following first-generation DES implantation, the differential vascular response between SES and PES, and characteristics of neoatherosclerosis following first-generation DES as compared to bare metal stents (BMS) in human coronary arteries.

## 2. Endothelial Coverage: The Most Important Morphometric Predictor for Late/Very Late Stent Thrombosis

To determine the pathologic correlates of late and very late ST following DES implantation, we investigated a total of 62 coronary lesions from 46 human autopsy cases with first-generation DES implanted for greater than 30 days [11]. We identified ST in 28 lesions (14 SES and 14 PES lesions from 23 patients) and compared those to 34 lesions (18 SES and 16 PES lesions from 23 patients) of similar duration without ST (duration of implant: 254 ± 235 days for lesions with late/very late ST versus 244 ± 289 days for those without, *P* = NS).

We found that neointimal thickness was less in thrombosed DES lesions (median 0.074 interquartile range [0.033,  0.129] versus patent DES: 0.11 [0.071, 0.19] mm, *P* = 0.05), and the percentage of endothelialization was significantly less in thrombosed DES lesions as compared to patent DES lesions (40.5 ± 29.8% versus 80.0 ± 25.2%, *P* < 0.0001). Total stent length was longer in thrombosed versus nonthrombosed stents (25.9 ± 11.5 versus 20.3 ± 9.6 mm, *P* = 0.04), and an average stent length without neointimal coverage was significantly greater in thrombosed as compared to nonthrombosed lesions (20.1 ± 11.5 versus 9.9 ± 10.1 mm, *P* = 0.0004). The mean number of uncovered struts per section was also significantly greater in DES lesions with thrombosis versus those without (5.0 ± 2.7 versus 2.0 ± 2.7, *P* < 0.0001), and the ratio of uncovered to total struts per section was greater in thrombosed versus nonthrombosed lesions (0.50 ± 0.23 versus 0.19 ± 0.25, *P* < 0.0001).

**Figure 1:**
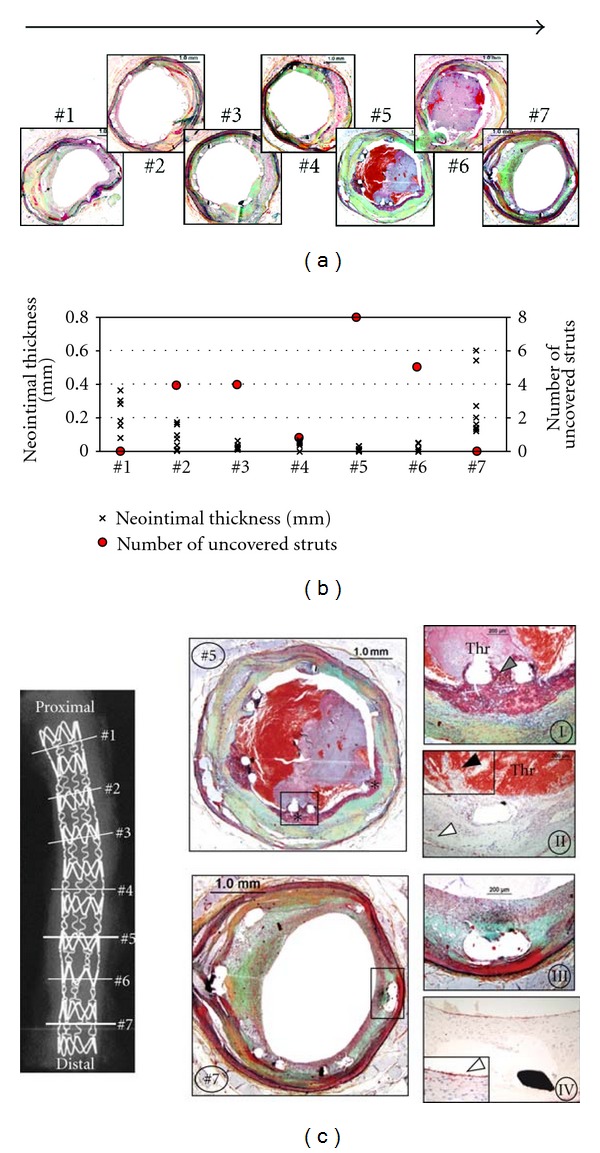
Heterogeneity of neointimal healing following drug-eluting stent placement. A 34-year-old woman underwent placement of one sirolimus-eluting stent (SES) (22 × 3 mm) stent in the proximal left circumflex artery for acute myocardial infarction 2 years antemortem. The patient was admitted to emergency room with ST-elevation myocardial infarction and subsequently expired. Consecutive sections of SES were cut 3 mm apart, stained with Movat Pentachrome (a), and measurements of neointimal thickness (above each strut) and the number of uncovered stent struts performed (b). There is greater neointimal growth above each strut (×) and fewer uncovered stent struts (red circle) within the proximal and distal stented portion, with absence of luminal thrombus formation. At the site of thrombus formation (Sections 5 and 6), neointimal thickness is minimal and the number of uncovered stent struts is maximal. (c), Movat Pentachrome-stained. Sections 5 and 7 show detailed histology. There is a platelet-rich thrombus surrounding stent struts lacking neointima (∗) in Section 5. High power images (I, II) show uncovered stent struts with extensive underlying fibrin deposition (grey arrowhead), luminal platelet-rich thrombus (Thr), and lack of endothelialization (II, black arrowhead) following immunostaining for CD31/CD34. However, positive staining (brown) is observed within medial microvessels containing CD31/CD34-positive endothelial cells (white arrowhead). In contrast, there is a well-healed neointima with complete strut coverage in Section 7. High power images (III, IV) show stent struts embedded into neointima composed of smooth muscle cells and proteoglycans; there is an absence of luminal thrombus, and endothelial cells are abundant above stent struts (IV, white arrowhead) with positive staining (brown). (Reproduced with permission from [11].)

Moreover, the average distance between individual stent struts was significantly shorter in DES lesions with thrombus formation as compared to patent DES lesions (0.52 ± 0.24 versus 0.70 ± 0.25 mm, *P* = 0.004). There was also a good correlation between the mean number of uncovered struts per section and the average distance between stent struts (*r* = −0.41, *P* = 0.001), with the majority of uncovered stent struts showing less interstrut distance than covered stent struts. On further examination, we found heterogeneity of coverage of stent struts, both within individual cross-sections as well as between sections from the same stent. Within the same DES, while some struts show healing as demonstrated by neointimal growth, others remained bare and serve as a nidus for thrombus formation (Figure 1). Within a DES, the middle sections of the stent (versus the proximal and distal ends) were the most common location of stent struts lacking neointimal coverage and this was also the most common site of thrombus formation.

Multivariable logistic-generalized estimating equations modeling demonstrated that endothelialization was the best predictor of late/very late ST. The morphometric parameter that best correlated with endothelialization was the ratio of uncovered to total stent struts per section. In a stent with greater than 30% uncovered struts, the odds ratio (OR) for thrombosis was 9.0 (95% confidence interval [CI]: 3.5 to 22.0) as compared to a stent with complete coverage.

The mechanisms by which the first-generation DES induces nonuniform incomplete healing are not fully understood; however, lesion characteristics, drug properties, total dose, release profile and drug distribution, and polymer biocompatibility, all play an important role in inducing neointimal suppression. While sirolimus and paclitaxel reduce neointimal formation by decreasing smooth muscle cell proliferation and migration, these drugs also impair the normal healing process of the injured arterial wall [13–15]. Underlying plaque morphology also affects the rate of healing when stent struts penetrate deeply into a necrotic core [16]. There is plaque prolapse with lack of cellular areas and a failure to form a healed neointima. Eccentric plaques may prevent uniform strut deployment thereby increasing local toxicity due to higher concentrations of drug and polymer. Indeed, sections with evidence of thrombosis showed significantly lower interstrut distances and this correlated with lower neointimal growth. Local concentration of drug is ultimately highly dependent on spacing of stent struts and the variance in distance between struts will amplify differences in concentrations leading to biological effects [17]. Heterogeneity in loaded dose of drug varies from strut to strut and there may be greater retention of lipophilic drugs in different regions of plaque affecting arterial drug concentration and resulting in nonuniform healing [18]. Coating defects can explain some of these differences [19]. The relationship between local drug concentration and cellular repair is further clarified by data from overlapping versus nonoverlapping SES and PES which illustrate less coverage of stent struts in overlapping segments as compared to nonoverlapping stent struts in the rabbit iliac model [20].

In summary, the underlying pathology in cases of late/very late ST indicates incomplete stent struts coverage as the most important morphometric predictor of late/very late ST and is also the most powerful surrogate of endothelialization. Both plaque- and device-related issues play a role in promoting uneven healing.

## 3. Delayed Arterial Healing in First-Generation DES for AMI

Given that acute myocardial infarction (AMI) is one of the only clinical presentations in which PCI has been shown to decrease the risk of death as compared to medical therapy alone, the long-term outcomes after DES for AMI are of immense clinical importance [21, 22]. Using our autopsy database of patients dying after DES implantation with duration of implant greater than 30 days, we compared the vascular pathologic responses to DES implantation in patients receiving first-generation DES for AMI (*n* = 17) with that of patients receiving for stable angina (*n* = 18) [23]. Histologic sections were evaluated for the identification of culprit and nonculprit sites. Culprit sites in AMI were defined as the stented segments with underling presence of a necrotic core and a thin cap with ruptured fibrous cap, whereas culprit sites in patients with stable angina were selected as the sections with the largest underlying necrotic core and overlying thick fibrous cap (>100 *μ*m). We compared culprit sites in patients with AMI to those in patients with stable angina as well as to nonculprit sites within each stent.

**Figure 2:**
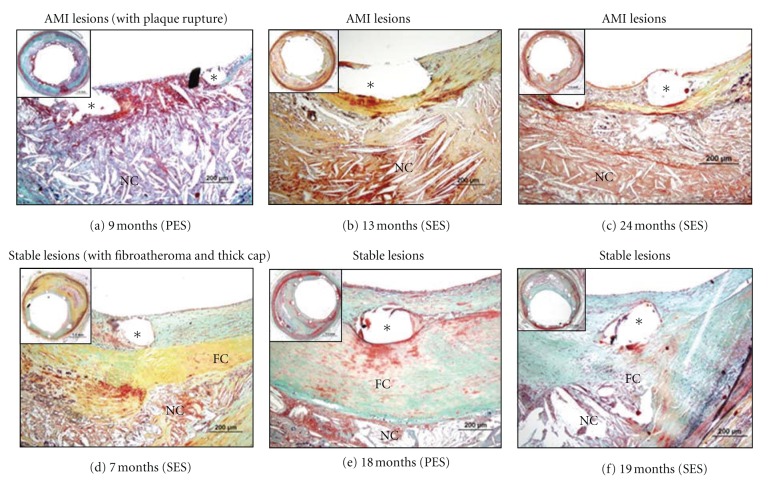
Histologic sections from patients with AMI and those with stable lesions. (a) through (c), Histologic sections from acute myocardial infarction (AMI) patients: a 64-year-old woman who died from congestive heart failure 9 months after paclitaxel-eluting stent (PES) implantation (a), a 49-year-old man who died from noncardiac cause 13 months after sirolimus-eluting stent (SES) implantation (b), and a 34-year-old woman who died from late stent thrombosis 24 months after SES implantation (c). Struts with necrotic core (NC) were observed with fibrin deposition and absence of endothelial coverage (a). Stents in (b and c) showed minimal coverage of struts above necrotic core at 13 or 24 months' duration. (d) through (f), Histologic sections from patients with stable lesions: a 61-year-old man who died from a noncardiac cause 7 months after SES implantation (d), a 53-year-old man who died suddenly 18 months after PES implantation (e), and a 68-year-old man who died from a noncardiac cause 19 months after SES implantation (f). All patients had underlying fibroatheroma with thick fibrous cap. High magnification images show underlying necrotic core (NC) and thick fibrous-cap (FC) with varying degree of neointimal formation above stent struts ((d) through (f)). (Reproduced with permission from [23.)

The incidence of late/very late ST was significantly higher in patients with AMI (7 of 17, 41%) as compared to those with stable angina (2 of 18, 11%; *P* = 0.04). Very late ST (>1 year) was observed in 2 patients with AMI (12%) and in no patients with stable angina. Morphometric analysis showed that culprit AMI sites versus stable plaque had significantly less neointimal thickness (0.04 [0.02, 0.09] versus 0.11 [0.07, 0.21] mm, *P* = 0.008), greater fibrin deposition (63 ± 28% versus 36 ± 27%, *P* = 0.008) and inflammation (35 [27, 49]% versus 17 [7, 25]%, *P* = 0.003), and higher prevalence of uncovered struts (49 [16, 96]% versus 9 [0, 39]%, *P* = 0.01). Representative images of culprit sites from patients with AMI and those with stable angina are illustrated in Figure 2.

In patients with AMI, neointimal thickness was significantly less at culprit sites as compared to nonculprit sites (0.04 [0.02, 0.09] versus 0.07 [0.04, 0.20] mm, *P* = 0.008), whereas this difference was not observed in patients with stable angina (0.11 [0.07, 0.21] versus 0.11 [0.08, 0.19] mm, *P* = 0.56). Similarly, the percentage of struts with fibrin (63 ± 28% versus 52 ± 27%, *P* = 0.04), struts with inflammation (35 [27, 49]% versus 30 [13, 38]%, *P* = 0.04), and uncovered struts (49 [16, 96]% versus 19 [3, 34]%, *P* = 0.02) was significantly greater at the culprit sites as compared to nonculprit sites in patients with AMI, whereas there were no significant differences in these parameters in patients with stable angina. We found that fibrous cap thickness correlated negatively with the presence of uncovered struts (*R* = −0.60, *P* = 0.0006), while there was a significant positive correlation between fibrous cap thickness and neointimal thickness (*R* = 0.68, *P* = 0.0001).

These findings reinforce our previous report demonstrating heterogeneity of healing within the same stent [11]. Plaque rupture is the most frequent cause of AMI [24], and this underlying plaque morphology is the most reasonable explanation for the delayed healing at culprit sites in AMI as opposed to nonculprit sites as well as culprit sites from stable patients. Since sirolimus and paclitaxel are highly lipophilic [18], it is likely that these agents have high affinity for lipid-rich plaques (i.e., necrotic core) and the drug dwells there for longer periods of time because of greater strut penetration as compared to when struts are exposed to adjacent inflamed regions of the plaque. In addition, the lipid-rich necrotic cores are avascular and have fewer smooth muscle cells within the fibrous cap. Therefore, these areas are less likely to be covered by migrating and proliferating cells from adjacent regions. Higher drug concentrations in these areas may also heavily influence healing by retarding smooth muscle cell proliferation as well as endothelial regrowth. In addition, thrombus burden may also play a role by increasing uptake of drug by the thrombus as shown by Hwang et al. with paclitaxel-eluting stents [25].

In randomized controlled trials enrolling patients with ST-elevation AMI (STEMI) treated with either BMS or DES, the majority of the long-term (3-4 years) follow-up data showed that DES had a lower incidence of target lesion revascularization with similar mortality and comparable incidence of ST as compared to BMS [26–28]. However, 5-year follow-up results of the PASSION study showed that the incidence of very late definite ST was significantly greater in PES as compared to BMS (3.3% versus 0.7%, *P* = 0.04), whereas the occurrence of cardiac death, recurrent myocardial infarction, and target lesion revascularization was comparable between PES and BM [7]. Furthermore, a recent meta-analysis of 15 randomized controlled trials comparing first-generation DES with BMS in patients with STEMI showed that an early benefit of DES in reducing target vessel revascularization was offset in subsequent years by an increased risk of very late ST [29]. During the first year following stent placement, patients with DES had a trend toward less definite ST as compared to those with BMS (relative risk: 0.80, 95% CI 0.58 to 1.12), whereas the risk of definite ST during subsequent years was significantly greater in DES as compared to BMS (relative risk: 2.10, 95% CI 1.20 to 3.69) [29].

Greater incidence of very late ST in STEMI patients with DES as compared to those with BMS has been further supported by large observational studies. Brodie et al. [30] reported the results from a large single-center registry enrolling consecutive patients receiving DES (SES, PES, or a second-generation DES [zotarolimus- or everolimus-eluting stents]; *n* = 368) or BMS (*n* = 1095) for STEMI, where the rate of definite/probable ST was similar between DES and BMS at 1 year (4.0% versus 5.1%) but increased more with DES after the first year (DES; 1.9%/year, BMS; 0.6%/year). Landmark analysis (>1 year) revealed that DES had a significantly greater incidence of very late ST (*P* < 0.001) and reinfarction (*P* = 0.003), and DES was the only independent determinant of very late ST (HR: 3.79, 95% CI: 1.64 to 8.79, *P* = 0.002). These findings are reminiscent of previous long-term registries, in which very late ST after primary PCI was only noted with DES [31, 32]. The results from there clinical studies showing greater incidence of very late ST in AMI patients treated with first-generation DES as compared to those with BMS are in line with our pathologic findings. On the contrary, a recent prospective registry-based study in STEMI showed that patients treated with DES (*n* = 200) had a significantly lower mortality as compared to those with BMS (*n* = 1369) at 3 years (DES: 4.2% versus BMS: 13.5%, *P* = 0.007), although the use of DES was not an independent determinant of all-cause mortality (adjusted OR: 0.5, 95% CI: 0.2 to 1.2, *P* = 0.10) [33]. Thus, clinical safety and efficacy of DES in patients with STEMI are still controversial.

In summary, vessel healing at culprit sites in AMI patients treated with first-generation DES is substantially delayed as compared to nonculprit sites and culprit sites in patients receiving DES for stable angina, emphasizing the importance of underlying plaque morphology in arterial healing and the risk of late/very late ST following DES implantation.

## 4. Pathologic Findings in Bifurcation Stenting

Atherosclerotic lesions tend to form at specific regions of the coronary vasculature where flow is disturbed, in particular in areas of low shear [34–36]. Because dramatic hemodynamic alternations occur at branch points within the arterial tree, coronary bifurcations are extraordinarily susceptible to atherosclerosis. Indeed, our human pathologic data in nonstented coronary bifurcation lesions showed that low shear area (the lateral wall) had significantly greater intimal thickness and necrotic core size as compared to high shear area (the flow divider) [37]. The use of DES at bifurcation lesions has reduced restenosis rates as compared to BMS; however, long-term outcomes are tempered by an increased risk of ST [38], which raises the possibility that delayed healing seen after DES implantation might be exacerbated at bifurcation sites. Given the difference in atherosclerotic plaque burden between low and high shear regions, neointimal growth following stent implantation may be different between these lesions. To investigate this hypothesis, we evaluated the pathologic arterial response to bifurcation stenting with DES and BMS [37].

**Figure 3:**
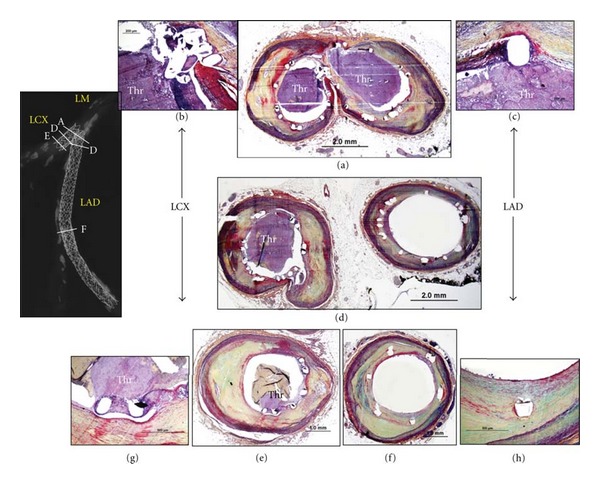
A case of late drug-eluting stent thrombosis in bifurcation lesion. A 55-year-old man with a history of smoking, hypertension, and dyslipidemia received 2 paclitaxel-eluting stents (PES) in the distal left main (ostium of left anterior descending coronary artery [LAD] and left circumflex [LCX]) with overlapping Taxus stents placed in the LAD. The patient died suddenly 2 years after stent implantation. Radiograph shows mildly calcified coronary artery. Both stents are occluded with platelet-rich thrombus (Thr) at the ostium of LAD and LCX (a). High magnification images demonstrated adherent thrombus in the region of the uncovered struts at the flow divider (b, c). Uncovered struts and adherent thrombus were also observed in all sections of the LCX stent (d, e, and g). The middle to distal portion of the LAD stent showed absence of thrombus and healed luminal surface with mild neointimal thickening (d, f, and h). (Reproduced with permission from [37].)

From our stent registry, a total of 40 stented bifurcation lesions (DES = 19 and BMS = 21) from 40 patients were reviewed. Duration of implant was similar between the DES and BMS groups (330 [188, 680] versus 150 [54, 540] days, *P* = 0.14). To assess the impact of flow disturbance on arterial healing in stented lesions, the differences between high shear (flow divider) and low shear (lateral wall) regions were compared. Neointimal thickness was significantly less at the high shear site as compared to the low shear in DES (0.07 [0.03, 0.15] versus 0.17 [0.09, 0.23] mm, *P* = 0.001), whereas this difference did not reach statistical significance for BMS cases (0.26 [0.16, 0.73] versus 0.44 [0.17, 0.67] mm, *P* = 0.25). Similarly, the percentage of uncovered struts was significantly greater at high shear as compared to low shear in DES (40 [16, 76]% versus 0 [0, 15]%, *P* = 0.001), while there was no significant difference in BMS (0 [0, 21]% versus 0 [0, 0]%, *P* = 0.09). Fibrin deposition was also frequently higher at sited of high shear as compared to low shear and was only observed in DES (60 [21, 67]% versus 17 [0, 55]%, *P* = 0.01). Although difference remained of borderline significance because of limited sample size, a greater incidence of late/very late ST was documented in the DES group as compared to the BMS group at bifurcation sites (main vessel: 75% versus 36%, *P* = 0.06; side branch: 42% versus 14%, *P* = 0.19). Interestingly, most of the thrombi originated at the flow divider site where uncovered struts were frequently observed (Figure 3).

Previous experimental study has shown that a greater neointimal formation occurs in the lateral as compared to flow divider region following stent implantation in porcine ilio-femoral bifurcation model [39], which is consistent with our findings. On the other hand, these differences were not significant in BMS, which may be due to more rapid healing and more uniform neointimal formation after BMS implantation. Thus, a combination of drug effect and blood flow disturbance is likely to accelerate the delayed healing in bifurcation lesion.

In summary, arterial healing at bifurcation lesions with first-generation DES was impaired with greater delay at the flow divider (high shear) as compared to the lateral wall sites (low shear), which may be caused by a combination of drug effect and difference in flow condition.

## 5. Impact of Stent Fracture on Adverse Pathologic Findings

Stent fracture has emerged as an important complication following DES implantation and is recognized as one of the contributors of in-stent restenosis [40–42] and ST [43, 44]. Clinically the incidence of stent fracture is reported as 1-2% of patients at 8 to 10-month follow-up on angiography [40, 45] however, the sensitivity of angiography to detect stent fracture is limited. We sought to assess the incidence of stent fracture at autopsy using high-contrast film-based radiography and investigated the impact of stent fracture on the pathologic findings and clinical outcomes [46].

High-contrast film-based radiographs of consecutive 177 lesions (SES = 77 and PES = 101, 1 lesion had both SES and PES) from our autopsy registry were reviewed. Stent fracture was graded as I (single strut fracture), II (2 or more strut fractures without deformation), III (2 or more strut fractures with deformation), IV (multiple strut fractures with acquired transection but without gap), and V (multiple strut fractures with acquired transection with gap in the stent body). The incidence of adverse pathologic findings (thrombosis and restenosis) was assessed histologically.

Stent fracture was documented in 51 lesions (29%; Grades I = 9, II = 14, III = 11, IV = 6, and V = 9). There was no significant difference in age, gender, and cause of death between patients with fracture and those without. Lesions with stent fracture had longer duration of implant as compared to those without fracture (172 [31–630] versus 44 [7–270] days, *P* = 0.004), whereas no statistical difference in duration of implant was identified between each grade of stent fracture (Grades I; 31 [5, 616], II; 105 [27, 1095], III; 376 [72, 570], IV; 331 [31, 833], and V; 172 [44, 450] days, *P* = 0.70). Furthermore, lesions with stent fracture showed a higher rate of SES usage (63% versus 36%, *P* = 0.001), longer stent length (30.0 [22.0–40.0] versus 20.0 [14.0–27.3] mm, *P* < 0.0001), and a higher rate of overlapping stents (45% versus 22%, *P* = 0.003) as compared to lesions without stent fracture. A forward stepwise logistic regression analysis demonstrated that longer stent length (OR: 1.07, 95% CI: 1.036 to 1.100, *P* < 0.0001), use of SES (OR: 3.40, 95% CI: 1.57 to 7.33, *P* = 0.002), and longer duration of implant (OR: 1.002, 95% CI: 1.001 to 1.003, *P* = 0.002) were independent determinants of stent fracture.

**Figure 4:**
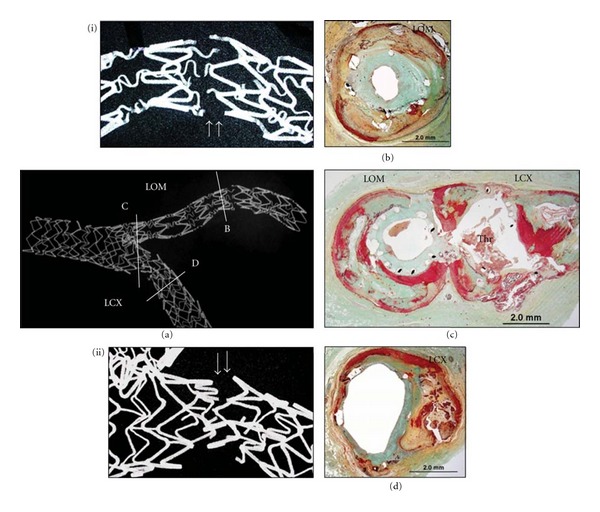
A case of grade V stent fracture: SES and PES. A 68 year-old woman died suddenly from stent thrombosis in left circumflex artery (LCX) 172 days following stent implantation. Radiograph showing the stented LCX and left obtuse marginal artery (LOM) (a). Note, presence of grade V sirolimus-eluting stent (SES) fracture highlighted in magnified image (i) and another grade V fracture at the bifurcation site in the paclitaxel-eluting stent (PES) (ii). SES in LOM with grade V fracture was associated with restenosis (b). PES fracture in LCX was located in the area close to the bifurcation site where the thrombus was located (Thr). The stented LCX segment distal to the fracture was widely patent (d). (Reproduced with permission from [46].)

Histologic evaluation showed that neointimal thickness was similar between lesions with stent fracture and those without (0.11 [0.06, 0.19] versus 0.11 [0.03, 0.19] mm, *P* = 0.62). There was no significant difference in fibrin deposition (fibrin score: fracture (+); 1.0 [0.1, 1.5] versus fracture (−); 1.4 [0.4, 2.0]) and inflammation (inflammatory score: fracture (+); 1.0 [0.5, 1.6] versus fracture (−); 1.4 [0.4, 2.0]) including similar degree of giant cell and eosinophil infiltration. Furthermore, there were no differences in these parameters among various fracture grades (i.e., grade I–V). Six adverse events (5 thrombosis and 1 restenosis) were associated with grade V fracture (67%), while there were no fracture site-related adverse pathologic findings in stents with grades I to IV fracture except for one stented lesion with grade IV which had a long overlapping stent (grade I–IV versus grade V, *P* < 0.0001). Representative case of grade V fracture of DES is shown in Figure 4.

Although it is not fully understood why stent fractures cause adverse events, the lack of stent integrity such as distortion or acquired under expansion may play an important role for the occurrence of adverse event. Previous clinical studies have reported that the main risk factors for stent fracture are longer stent length, RCA or saphenous vein graft lesion location, lesion with high motion, overlapping stent, and SES use [40, 42, 47]. Our findings showed that SES was associated with higher incidence of stent fracture; the flexible “N” shaped undulating longitudinal intersinusoidal-ring linker segment was the most frequent location of the fractures, which are smaller in width than the sinusoidal-ring portion. The relation between longer implant duration and higher incidence of stent fracture suggests that stent fracture may result from continuous stress over time to the implant which leads to greater metal fatigue with eventual fracture. However, it should also be noted that stent fracture was seen even in the patients who died shortly after stent implantation, which is probably procedure related (high pressure and/or oversized balloon, overlapping stent, etc.).

In summary, the incidence of DES fracture was 29% of the stented lesions at autopsy, which is much higher than clinically reported. A high rate of adverse pathologic findings was observed in lesions with grade V fracture, while fracture with grade I–IV did not have significant impact on the clinical outcome. Longer stent length, SES usage, and longer duration of stent implant were identified as independent predictors of stent fracture.

## 6. Coronary Responses and Differential Mechanisms of Late/Very Late Stent Thrombosis Attributed to SES and PES

Previous clinical trials have reported differences in angiographic late lumen loss in patients receiving SES or PES [48], whereas it remains unclear whether the long-term histologic responses to each stent type are different and how this relates to the time course of arterial healing and mechanism(s) of late/very late ST. Therefore, we evaluated vascular healing response and the mechanism(s) of late/very late ST in patients with first-generation DES [49]. Comparison of vascular response following SES and PES placement was further performed based on “on-label” or “off-label” indication. The “off-label” use was defined as stents deployed for AMI or bifurcation lesions, left main, bypass graft, restenosis, chronic total occlusion, or lesion lengths >30 mm [50]. The overall analysis included 174 cases (230 DES lesions) from our autopsy registry, and histomorphometry was performed on coronary stents from 127 cases (171 lesions) who died ≥30 days after receiving stent implants. Analysis of individual lesions with duration of implant <30 days (SES = 25 and PES = 34) revealed that the incidence of early ST was equivalent for lesions with SES and PES (44% versus 38%, *P* = 0.79). Histologically, no differences in the extent of inflammation and fibrin deposition were noted between SES and PES implants <30 days.

Lesions with duration of implant ≥30 days comprised of 77 SES and 94 PES lesions where 40 SES (52%) and 53 PES (56%) lesions were treated for “off-label” indications. Neointimal thickness was significantly greater in PES as compared to SES (0.13 [0.03, 0.20] versus 0.10 [0.04, 0.15] mm, *P* = 0.04). Similarly, PES had greater maximum neointimal thickness than SES (0.23 [0.13, 0.37] versus 0.17 [0.06, 0.28] mm, *P* = 0.04). The heterogeneity in neointimal thickness between sections was also significantly greater for PES versus SES (0.14 [0.08, 0.31] versus 0.10 [0.03, 0.22] mm, *P* = 0.02). On the other hand, the percentage of uncovered struts was similar between PES and SES (20% versus 21%, *P* = 0.72). There was a progressive and significant increase in neointimal thickness beyond 9- and 18-month duration in lesions with PES without evidence of late/very late ST (*P* = 0.009). Similar trends were observed for SES with borderline significance (*P* = 0.08).

Accumulated fibrin as assessed by fibrin score was significantly greater in PES as compared to SES (1.8 [1.0, 2.5] versus 0.8 [0.0, 2.0], *P* = 0.001). On the contrary, SES implants were associated with a significantly greater inflammatory score as compared to PES (1.3 [0.5, 2.0] versus 1.0 [0.5, 1.5], *P* = 0.007). The contributing cells resulting in greater inflammation observed with SES were predominantly eosinophils and giant cells. The incidence of malapposition was comparable between SES and PES (14% versus 19%, *P* = 0.40), although the underlying mechanism was different (see below). A further analysis revealed near complete healing in stents placed for “on-label” indications with implant durations of >12 months, while the majority of DES with “off-label” usage remained unhealed beyond similar time point.

**Figure 5:**
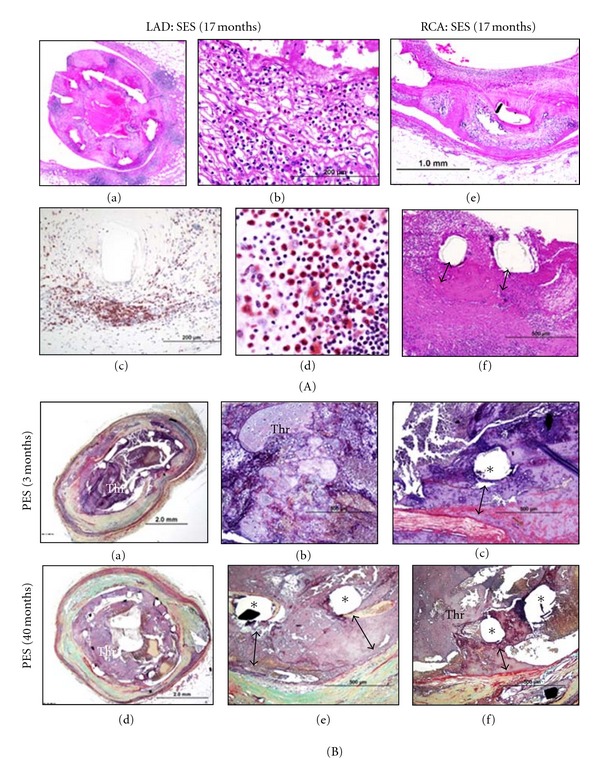
Representative images of late/very late stent thrombosis in SES and PES. (A) Histologic sections from sirolimus-eluting stents (SES). A 40-year-old woman who received 2 SES in left anterior descending artery (LAD) and right coronary artery (RCA) 17 months antemortem died suddenly 4 days after surgical removal of melanoma (wide excision). Antiplatelet therapy (aspirin and clopidogrel) was discontinued 5 days before the surgery. Histologic sections of the SES in LAD showed total thrombotic occlusion and diffuse inflammation (a). Numerous inflammatory cells were observed within neointimal area (b). Inflammatory reaction predominantly consists of T-lymphocytes (c) (CD45Ro) and eosinophils (d) (Luna stain). Note, the same reaction was observed in the SES in RCA (e) and severe inflammation resulted in malapposition of stent struts (f). (B) Histologic sections from paclitaxel-eluting stent (PES) showing malapposition. A 69-year-old man who received a PES in a saphenous vein graft died suddenly 3 months after the stent placement. Histologic sections showed thrombotic occlusion in the PES (a, b); note the malapposition secondary to severe fibrin deposition (c). A 48-year-old man with PES implantation in the proximal LAD died suddenly at 40 months. Histologic sections showed thrombotic occlusion of the PES (d). Most struts are malapposed with fibrin deposition underneath the stent struts (e and f). Thr = thrombus. (Reproduced with permission from [49].)

The incidence of late/very late ST did not differ significantly between SES and PES (SES = 21% [16/77] versus PES = 27% [25/94], *P* = 0.47) (Table 1). Underlying pathologic causes of late/very late ST was determined as penetration of necrotic core, bifurcation stenting, long/overlapping stents, stent underexpansion, isolated uncovered struts, localized hypersensitivity reaction, and malapposition from excessive fibrin deposition (Table 1). The “procedure-related” causes of late/very late ST, including bifurcation stenting, long/overlapping stents, and underexpansion, were greater in PES as compared to SES (PES = 48% [12/25] versus SES = 13% [2/16], *P* = 0.04). On the other hand, differential vascular response to stents as underlying causes of late/very late ST was identified between SES and PES; for SES, there were localized hypersensitivity reactions, consisting of eosinophils, lymphocytes, and giant cells throughout the stented segment (Figure 5(a)), while late/very late ST in PES was attributed to malapposition secondary to excessive fibrin deposition on the abluminal surface (Figure 5(b)). The majority of patients with hypersensitivity reaction following SES implantation died in the very late phase (>1 year) where the mean duration of implant was 649 days. Malapposition was observed in 5 lesions (71%) with a mean section of struts from the vessel wall of 944 *μ*m. In most SES with severe inflammation, there was positive remodeling of the vessel resulting in a malapposition. In contrast, malapposition secondary to excessive strut fibrin as the primary contributor towards late/very late ST was observed only in PES with mean implant duration of 611 days. The mean distance separating the struts from the vessel wall was 404 *μ*m. The luminal surface generally lacked endothelial cell coverage as well as evidence of granulation tissue consisting of macrophages, smooth muscle cell, or proteoglycan matrix (Figure 5(b)).

As previously described, mechanism(s) of late/very late ST is likely multifactorial [10] While arterial healing response following DES placement is responsible for late/very late ST, clinical factors such as patient characteristics, interventional procedure competency, and patient adherence to medical treatment also contribute to adverse events. Although there is a certain commonality in the mechanism of late/very late ST for both SES and PES where the majority of the cases demonstrated poor endothelialization, our findings indicate that the final stimulus for thrombus development may be different based upon DES type. The disparities in vascular responses regarding the stent milieu are undoubtedly attributable to differences in drug, polymer coating, and unique elution profile for each device. The hypersensitivity reaction observed in SES is likely attributed to the polymer rather than drug [51], which is presumably completely eluted by 3 months. Preclinical DES implants in porcine coronary arteries showed escalating amounts of inflammation over time [52], which is consistent with our findings showing that the majority of hypersensitivity cases were documented in devices implanted for >1 year. Greater fibrin accumulation around struts in PES also remains consistent with prior preclinical findings which showed a dose-dependent increase in fibrin deposition and medial necrosis following deployment of PES in rabbit iliac arteries [53] as well as similar dose escalatory findings in a porcine model [54]. Therefore, we believe that paclitaxel itself is responsible for excessive fibrin deposition.

Clinical studies utilizing intravascular ultrasound (IVUS) in patients with first-generation DES have demonstrated that incomplete stent apposition and positive vessel remodeling are prevalent in patients with very late ST [55]. Histopathology of aspirated thrombus from cases with very late ST showed inflammatory cell infiltrates where eosinophils were more commonly observed in SES than PES, and eosinophil count correlated positively with incomplete stent apposition cross-sectional area and with expansive vessel remodeling as assessed by IVUS [56]. Moreover, coronary angiographic studies have demonstrated that late acquired “peristent contrast staining” (PSS; contrast staining outside the stent contour) was identified in 2.5% of patients who received SES, and the presence of PSS within 12 months following SES placement has been shown to be associated with subsequent risk of very late ST [57]. A recent optical coherence tomography (OCT) study in patients with SES has shown that PSS is characterized by incomplete stent apposition and the presence of multiple cavities between and outside the struts (named “multiple interstrut hollows” [MIH]) [58]. These clinical findings indicate that late acquired incomplete stent apposition with positive vessel remodeling and MIH are suggestive of hypersensitivity reaction to SES.

Since histologic sections of PES demonstrating the thinnest neointima are typically accompanied by persistent fibrin, the heterogeneity of arterial healing may result from an uneven distribution of drug and polymer. Further support for variations of available paclitaxel within a single stent is from scanning electron microscopy (SEM) studies demonstrating webbing and delamination of polymer, which is a frequent finding in PES [19]. Progressive neointimal growth, although slow to develop, is likely related to persistent fibrin and inflammation. In both DES platforms, biological signs of a drug effect such as fibrin remain beyond the reported durations of drug release. Fibrin degradation products, in particular fibrin fragment E, are known initiators of smooth muscle cell migration and proliferation [59, 60], a phase that generally occurs early after BMS placement. In addition to fibrin, persistent inflammation is yet another plausible explanation for the late increase in neointimal formation associated with DES. Nonerodible polymers used in first-generation DES are associated with chronic inflammation and, in particular for SES, eosinophils, lymphocytes, and giant cells especially in the presence of hypersensitivity vasculitis [10, 20, 51, 61]. However, hypersensitivity cases failed to show an increase in neointimal thickness. Clinical studies have shown that target vessel revascularization rates do increase with time [62–64], and gradual growth of neointima seen in our study may partly account for this phenomenon. Moreover, greater prevalence of unhealed stents in DES placed for “off-label” as compared to “on-label” indications may have implications for the duration of dual antiplatelet therapy after first-generation DES placement.

In summary, first generation DESs exhibit divergent mechanisms of late/very late ST where hypersensitivity likely plays a significant role in SES while for PES, the etiology appears to be association with excessive fibrin deposition on abluminal surface with malapposition. Another important finding was near complete healing in DES placed for greater than 12 months with confirmed “on-label” usage, while “off-label” indications of both stents resulted in incomplete healing even in DES beyond 12 months.

## 7. The Pathology of Neoatherosclerosis in Human Coronary Implants: BMS versus DES

Atherosclerosis is characterized by the accumulation of lipid laden foamy macrophages in native coronary arteries, which develop over decades, whereas newly formed atherosclerotic changes within the neointima (neoatherosclerosis) following BMS and DES implantation occur much faster. The incidence, character, and temporal development of neoatherosclerosis occurring within BMS and DES at autopsy were examined where a total of 299 consecutive cases (142 BMS, 157 DES [81 SES and 76 PES] cases) with 406 lesions with duration of implant >30 days (197 BMS, 209 DES [103 SES and 106 PES] lesions) were reviewed [12]. Stent-related deaths from thrombosis were significantly more frequent in DES as compared to BMS (20% versus 4%, *P* < 0.001), whereas restenosis as a cause of death was more frequent in BMS than DES (28% versus 7%, *P* < 0.001). The median duration of implant was shorter in lesions treated with DES versus BMS (DES; 361 [172, 540] versus BMS; 721 [271, 1801] days, *P* < 0.001). Notably, 85% (177 lesions) DES were implanted for 2 years or less with no lesions extending beyond 6 years, while 45% (88 lesions) BMS were implanted for 2 years or less and 17% (33 lesions) had durations of more than 6 years. Stent lengths were significantly longer in DES as compared to BMS (22.0 [15.5, 30.0] versus 16.0 [12.0, 24.0] mm, *P* < 0.001), and the underlying plaque morphology was also different with unstable lesions (i.e., ruptured plaques and thin-cap fibroatheromas) more commonly found in DES as compared to BMS (*P* = 0.008).

**Figure 6:**
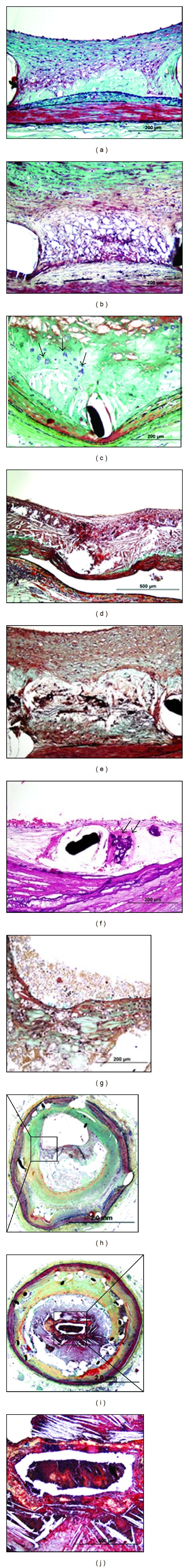
Representative images of the various stages of newly formed atherosclerotic changes within the neointima (neoatherosclerosis) following stent implantation. Foamy macrophage clusters in the peristrut region of sirolimus-eluting stents (SES) implanted for13 months antemortem is seen in (a). Fibroatheroma with foamy macrophage rich lesion and early necrotic core formation in SES of 13-month duration is shown in (b). Panel (c) shows fibroatheroma with peristrut early necrotic core, cholesterol clefts, surface foamy macrophages, and early calcification (arrow) in SES at 13 months. Peristrut late necrotic core in the neointima characterized by large aggregate of cholesterol cleft in SES at 17 months is shown in (d). Panel (e) shows fibroatheroma with calcification in the necrotic core in SES of 10-month duration. A peristrut calcification (arrow) with fibrin in SES of 7 months duration is shown in (f). (g and h) A low (h) and high power (g) magnification image of a severely narrowed bare metal stents (BMS) implanted 61 months with a thin-cap fibroatheroma. Note macrophage infiltration and a discontinuous thin fibrous cap (g). (i and j) A low magnification image shows a plaque rupture with an acute thrombus that has totally occluded the lumen in BMS implanted for 61 months antemortem (i). A high magnification image shows a discontinuous thin-cap with occlusive luminal thrombus (j). (Reproduced with permission from [12].)

**Figure 7:**
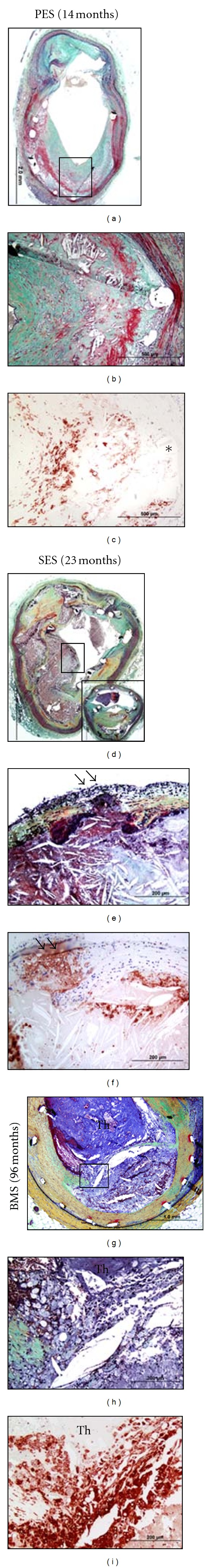
Representative cases showing atherosclerotic change following BMS, SES, and PES implantation. (a to c) Histologic sections from a 65-year-old woman with a paclitaxel-eluting stent (PES) implanted in the left circumflex artery 14 months antemortem, who died of traumatic brain injury. A low power image shows a patent lumen with moderate neointimal growth (a); foamy macrophage infiltration and necrotic core formation with cholesterol clefts are seen at high magnification in (b). (c) Same section as (b) showing CD68 positive macrophages in the neointima. (d to f) Histological sections from a 59-year-old male with sirolimus-eluting stents (SES) implanted for 23 months who died from stent thrombosis (d). Note thin-cap fibroatheroma with fibrous cap disruption in (e) (arrows) from boxed area in (d). The thrombus (Th) was more apparent in the distal section taken 3 mm apart (d). (f) shows CD 68 positive macrophages in the fibrous cap and in the underlying necrotic core. (g to i) Histologic section from a 47-year-old male who had a bare metal stents (BMS) implanted 8 years prior to death. Note occlusive thrombus (Th) in the lumen and ruptured plaque (boxed area in (g)), which is shown at higher magnification in (h) with large number of macrophages within the lumen as well as at the ruptured cap. Note large number of CD 68 positive macrophages at the site of rupture (i). (Reproduced with permission from [12].)

Representative images of various stages of neoatherosclerosis following stent implantation are shown in Figure 6. The earliest duration of implant associated with foamy macrophage accumulation was observed at 70 days for PES, 120 days for SES, and 900 days for BMS [12]. Necrotic core formation was identified early at 270 days for PES, 360 days for SES, and 900 days for BMS. Representative images of fibroatheroma with necrotic core from a PES implant of 14 months duration are illustrated in Figures 7(a) to 7(c). More advanced lesions—unstable features of neoatherosclerosis, namely, thin-cap fibroatheromas and in-stent plaque rupture (Figures 6(g) to 6(j) and 7(d) to 7(i))—were identified in both BMS (*n* = 7, 4%) and DES (*n* = 3, 1%), where the majority of BMSs were >5 years (average implant duration 6.1 ± 1.5 years) while for DES, unstable neoatherosclerotic lesions were identified with devices implanted ≤2 years (1.1, 1.4, and 1.9 years).

**Figure 8:**
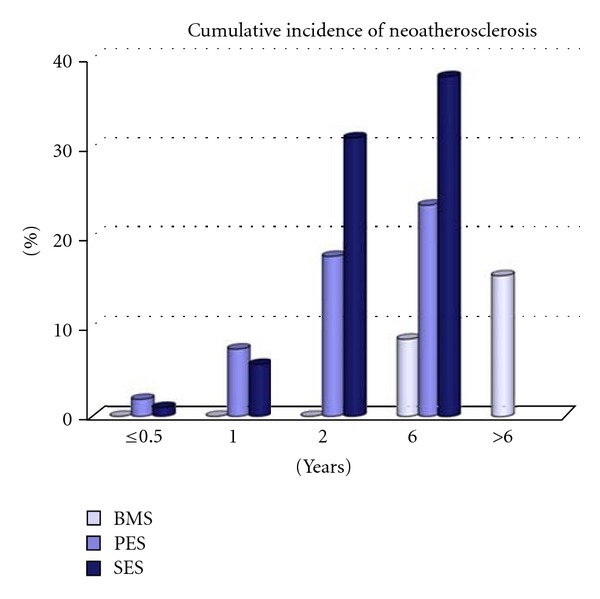
Bar graph showing cumulative incidence of atherosclerotic change with time following implantation of BMS versus SES, and PES. Both sirolimus-eluting stents (SES) and paclitaxel-eluting stents (PES) show earlier onset of neoatherosclerosis and a higher incidence of lesion formation as compared to bare metal stents (BMS). No drug-eluting stents (DESs) were available beyond 6 years. (Reproduced with permission from [12].)

The overall incidence of neoatherosclerosis was significantly greater in DES as compared to BMS (31% versus 16%, *P* < 0.001) despite longer duration of implant for BMS. The incidence of neoatherosclerosis was also evaluated based on the stratified duration of implant. For those of implants 2 years or less, DES had a greater incidence of any neoatherosclerosis (DES; 29% versus BMS; 0%, *P* < 0.001), which was represented by a greater incidence of foamy macrophage clusters (DES; 14% versus BMS; 0%, *P* < 0.001) as well as fibroatheromas (DES; 13% versus BMS; 0%, *P* < 0.001). For durations between 2 and 6 years, the DES group still expressed a higher incidence of neoatherosclerosis (DES; 41% versus BMS; 22%, *P* = 0.053). The incidence of any neoatherosclerosis was greater in SES than PES for duration of implant 2 years or less (SES; 37% versus PES; 21%, *P* = 0.021), although differences did not remain significant with stents implanted for 2 to 6 years (SES; 44% versus PES; 38%, *P* = 0.719). The cumulative incidence of any neoatherosclerosis with time after implantation of BMS versus SES and PES is shown in Figure 8. Neoatherosclerosis was observed more frequently and at earlier time point in first-generation DES as compared to BMS.

The risk factors for the development of neoatherosclerosis were evaluated by a multiple logistic generalized estimating equations modeling where younger age (OR: 0.963, 95% CI: 0.942 to 0.983, *P* < 0.001), longer duration of implant (OR: 1.028, 95% CI: 1.017 to 1.041, *P* < 0.001), SES usage (OR: 6.534, 95% CI: 3.387 to 12.591, *P* < 0.001), PES usage (OR: 3.200, 95% CI: 1.584 to 6.469, *P* = 0.001), and underlying unstable plaque (OR: 2.387, 95% CI: 1.326 to 4.302, *P* = 0.004) were identified as independent determinants.

The underlying processes responsible for the development of neoatherosclerosis following stent implantation are likely multifactorial; however, these involve the inability to maintain a fully functional endothelialized luminal surface within the stented segment. The endothelium normally provides an efficient barrier against the excessive uptake of circulating lipid, whereas the endothelial cells within the stented segment in DES show poorly formed intercellular junctions, reduced expression of antithrombotic molecules, and decreased NO production [65, 66]. Local inflammation induced by drugs and/or polymers may also be associated with activation of endothelial cells which express intercellular adhesion molecule-1 (ICAM-1) and vascular cell adhesion molecule-1 (VCAM-1) that attract circulating monocytes to the subendothelial space to convert to macrophages. Inefficient barrier of the endothelium within the stented segment is characterized by increased permeability with poor cell-to-cell contact as well as increased inflammation, which allow greater amount of lipoproteins to enter subendothelial space where matrix proteins such as proteoglycans promote their retention [67]. Retained lipoproteins in the subendothelial space further undergo oxidative modifications, which lead to production of chemoattractant and inflammatory mediators such as monocyte chemoattractant protein-1 (MCP-1) and VCAM-1, which are involved in the recruitment and attachment of monocytes [68]. Furthermore, stent-induced flow disturbances contribute to the complex spatiotemporal shear stress, which lead to the changes in endothelial phenotype (mechanotransduction) with increased expression of transmembrane proteins that further allow inflammatory cell attachment and migration to subendothelial spaces [69, 70].

In addition to the lipid uptake, macrophage and smooth muscle cell death may also dictate the development of neoatherosclerosis. The death of resident macrophages that particularly localize near stent struts may contribute to the pool of free cholesterol and cholesterol esters thereby forming a necrotic core [71]. Natural and/or drug-induced smooth muscle cell death may also yield free cholesterol and cholesterol esters, which may further attract macrophages [72]. Along similar lines, there is experimental evidence to suggest that neoatherosclerosis can occur within stents that is associated with delayed arterial healing compounded by lethal injury to smooth muscle cells and endothelial cells. Previous study demonstrated that a ^32^P *β*-emitting stent implanted with activities ranging from 6 *μ*Ci to 48 *μ*Ci showed focal evidence of neoatherosclerosis in normal arteries of New Zealand White rabbits examined at 6- and 12-months [73]. Considering it is well known that atherosclerosis does not develop in normal arteries of the rabbit in the absence of hypercholesterolemia, the atherogenic process is inherent to processes occurring around the stent itself.

In summary, in-stent neoatherosclerosis occurs in both BMS and DES; however, for DES implants, it is observed more frequently and at an earlier time point as compared to BMS. Moreover, neoatherosclerosis in DES shows unstable characteristics by 2 years following implant, while similar features in BMS occur at relatively later time points (average implant duration 6 years). The development of neoatherosclerosis is another important contributing factor for very late ST.

## 8. Conclusions

First-generation DES has dramatically reduced restenosis as compared to BMS; however, this comes at the price of increased risk of late and very late ST. Delayed arterial healing characterized by poor endothelialization has been identified as the primary pathologic substrate responsible for late and very late ST, which is associated with stent struts penetrating into the necrotic core (e.g., stenting for AMI lesions), long/overlapping stents, bifurcation stenting (especially in flow divider [high shear] region), hypersensitivity reaction (in SES), and malapposition with excessive fibrin deposition (in PES). Grade V stent fracture induces adverse pathologic findings including ST and restenosis. Uncovered struts decrease overtime but can be identified in both SES and PES with duration of implant beyond 12 months, particularly in stents placed for “off-label” indications. Neoatherosclerosis is another important contributing factor for very late ST, and DES shows more frequent and rapid development of neoatherosclerosis as compared to BMS. Future pathologic studies should investigate vascular response to newer generation DES including second-generation zotarolimus-(ZES) and everolimus-eluting stents (EES), biodegradable polymer coated and polymer free DES, and completely bioerodible scaffold, to clarify if these newer technologies contribute to the reduced risk of late/very late ST and better patient outcomes. 

## Figures and Tables

**Table 1:** Incidence and underlying causes of late/very late stent thrombosis in SES and PES.

	SES	PES	*P* value
Incidence of late/very late ST	16/77 (21%)	25/94 (27%)	0.47
Underlying causes of late/very late ST			
AMI, penetration of the necrotic core	5 (31%)	5 (20%)	0.47
Bifurcation stenting	1 (6%)	9 (36%)	0.06
Long/overlapping stents	0 (0%)	2 (8%)	0.50
Stent underexpansion	1 (6%)	1 (4%)	1.00
Isolated uncovered struts	2 (13%)	1 (4%)	0.55
Localized hypersensitivity	7 (44%)	0 (0%)	0.0005
Malapposition from excessive fibrin deposition	0 (0%)	7 (28%)	0.03

AMI: acute myocardial infarction; PES: paclitaxel-eluting stents; SES: sirolimus-eluting stents; ST: stent thrombosis (Reproduced with permission from [49].)
